# Progesterone Alleviates Neural Behavioral Deficits and Demyelination with Reduced Degeneration of Oligodendroglial Cells in Cuprizone-Induced Mice

**DOI:** 10.1371/journal.pone.0054590

**Published:** 2013-01-24

**Authors:** Jian-Ning Ye, Xing-Shu Chen, Le Su, Yun-Lai Liu, Qi-Yan Cai, Xiao-Li Zhan, Yan Xu, Shi-Fu Zhao, Zhong-Xiang Yao

**Affiliations:** 1 Department of Neurology, Xin Qiao Hospital, Third Military Medical University, Chongqing, China; 2 Department of Histology and Embryology, Third Military Medical University, Chongqing, China; 3 Squadron 9 of Cadet Brigade, Third Military Medical University, Chongqing, China; 4 Department of Physiology, Third Military Medical University, Chongqing, China; Hannover Medical School, Germany

## Abstract

Demyelination occurs widely in neurodegenerative diseases. Progesterone has neuroprotective effects, is known to reduce the clinical scores and the inflammatory response. Progesterone also promotes remyelination in experimental autoimmune encephalomyelitis and cuprizone-induced demyelinating brain. However, it still remains unclear whether progesterone can alleviate neural behavioral deficits and demyelination with degeneration of oligodendroglial cells in cuprizone-induced mice. In this study, mice were fed with 0.2% cuprizone to induce demyelination, and treated with progesterone to test its potential protective effect on neural behavioral deficits, demyelination and degeneration of oligodendroglial cells. Our results showed noticeable alleviation of neural behavioral deficits following progesterone treatment as assessed by changes in average body weight, and activity during the open field and Rota-rod tests when compared with the vehicle treated cuprizone group. Progesterone treatment alleviated demyelination as shown by Luxol fast blue staining, MBP immunohistochemical staining, and electron microscopy. There was an obvious decrease in TUNEL and Caspase-3-positive apoptotic cells, and an increase in the number of oligodendroglial cells staining positive for PDGFRα, Olig2, Sox10 and CC-1 antibody in the brains of cuprizone-induced mice after progesterone administration. These results indicate that progesterone can alleviate neural behavioral deficits and demyelination against oligodendroglial cell degeneration in cuprizone-induced mice.

## Introduction

Demyelination occurs widely in the central nervous system (CNS) during neurodegenerative diseases [1]. Progesterone has neuroprotective effects on brain and spinal cord injury, and neurodegenerative diseases [2]–[8]. Progesterone can increase remyelination in a demyelinating model, which has previously been induced by stereotaxic injection of ethidium bromide into the caudal cerebellar peduncles, in spinal cord injury, and in the spinal cord of mice with lysophospatidylcholine-induced demyelination [9]–[12]. Progesterone can promote oligodendroglial cell proliferation and the formation of new myelin sheaths [13], [14]. Progesterone can enhance the density of oligodendrocyte progenitor cells (OPCs) and induce their differentiation into mature oligodendrocytes [15], [16].

Multiple sclerosis (MS) is a typical demyelinating disease, and experimental autoimmune encephalomyelitis (EAE) is one of the most frequently used animal models of MS [17]–[19]. It has been reported that the rate of relapse of MS significantly declines during the third trimester of pregnancy, and significantly increases during the first three months post-partum. Progesterone is used to prevent the post-partum relapse in MS [17]. Progesterone also has beneficial effects in EAE mice, by delaying EAE onset, reducing the clinical scores and the inflammatory response to decrease demyelination, and decreasing the swelling and apoptosis of neural cells. Also, progesterone exerts protective effects on axonal damage in the spinal cord of EAE mice [4]–[6], [18]–[21].

The neurotoxicant cuprizone, a copper chelating molecule, has been used extensively to create a mouse demyelinated model and study the mechanisms of demyelination and remyelination [22], [23]. Progesterone can increase remyelination in the demyelinated brain. The combined application of estradiol and progesterone can protect the brain from demyelination in cuprizone-induced mice [24]. However, it still remains unclear whether progesterone can alleviate neural behavioral deficits and demyelination against degeneration of oligodendroglial cells in cuprizone-induced mice. In this study, we observed the protective effect of progesterone on neural behavioral deficits in cuprizone-induced mice by detecting changes in body weight, and activity during the open field and Rota-rod tests, as well as against demyelination *via* its inhibition of the degeneration of oligodendroglial cells.

## Materials and Methods

### Animals and Tissue Preparation

Male C57BL/6 mice were obtained from the Experimental Laboratory Animal Center of the Third Military Medical University. All experiments in this study were performed in accordance with protocols specifically approved (IACUC: 09446) by the Third Military Medical University Institutional Animal Care and Use Committee. Mice were sacrificed using pentobarbital sodium.

### Progesterone and Cuprizone Treatment

Six-week-old (15–17 g) male C57BL/6 mice were randomly divided into four groups (n = 25 in each group). Two groups were fed a normal diet, and the other two groups were fed diets containing 0.2% (w/w) cuprizone (Sigma) to induce demyelination. Progesterone (Sigma) was dissolved in dimethyl sulfoxide (DMSO). DMSO was used as the vehicle. Vehicle or progesterone (5 mg/kg) was subcutaneously injected into the neck of these mice with or without cuprizone treatment every other day for 2 weeks during the 2nd week to the end of the 3rd week. Thus, the four groups were named: vehicle-treated normal group (N+Veh), progesterone treated normal group (N+P), vehicle-treated cuprizone group (CPZ+Veh) and progesterone-treated cuprizone group (CPZ+P). These treated mice were sacrificed every week from the first week to the fifth week, and the majority of mice were sacrificed at the end of the fifth week. The plasma levels of progesterone reached approximately 50 ng/mL.

### Body Weight

The body weight of every test mouse was measured at 4∶00 in the afternoon every other day throughout the experimental period.

### Open Field Test

All the mice (n = 15 per group) were housed on a 12-h light cycle. Silence was maintained during the testing period. The open-field apparatus (Biowill, China) consisted of a 25 cm×25 cm plastic square surrounded by a 32-cm-high wall. The open-field test was administered at 4∶30 in the afternoon. The mice were placed into the apparatus at the beginning of the test, and allowed to move freely around the apparatus and explore the new environment. The total and center-area distances were measured by a video tracking program (Biowill, China). The paths of the mice were recorded for 5 min by a video camera (Digital CCD Camera, Sony, China) placed above the square.

### Rota-rod Test

Three trials were applied at 6∶00 in the afternoon for three consecutive days before the Rota-rod test began. The mice were placed on a rotating cylinder (YLS-4C, Biowill, China) that was accelerated from 5 to 40 revolutions per minute (rpm), and kept at 40 rpm. If the mice fell or remained on the cylinder for two cycles, the test was stopped. The total time the mice remained on the cylinder was calculated. All the mice were evaluated on the Rota-rod three times a day. The test was repeated after a 15-min rest. The length of time the mice stayed on the cylinder correlated with motor coordination and balance; longer times were equated with better coordination and balance.

### Myelin Staining by Luxol Fast Blue (LFB) and Cresyl Violet

Eight-micrometer paraffin sections were deparaffinized, then stained with LFB (Sigma) solution (0.01% LFB in 95% ethanol with 10% acetic acid) at 60°C for 18 h. These sections were hydrated with 95% and 75% alcohol, differentiated by 0.05% lithium carbonate solution for 30 s, then dipped in 70% ethanol, and stopped immediately with distilled water. Then the tissues were counterstained with cresyl-violet (Sigma) for 10 min at 37°C. The demyelination area lacked LFB staining in the white matter.

### Transmission Electron Microscope

All the mice were deeply anesthetized by intraperitoneal 100 mg/kg pentobarbital injections, perfused with 37°C normal saline and fixed with paraformaldehyde. The genu area of the corpus callosum was identified under a dissecting microscope, and was cut into the block from the mid-corpus callosum up to one-third of the splenum, according to the Plate 29–48 from the atlas of Franklin and Paxinos (2001) [25], then quickly fixed in 2.5% glutaraldehyde at 4°C for 24 h. The block was further cut into 1×1×3 mm sagittal fragment and post-fixed in 1% osmium tetroxide for 2 h. After dehydration with cold acetone, the fragment was embedded in araldite epoxy resin, cut sagittally in half and stained with toluidine blue. The fragment was cut into 60-nm ultra-thin sections, and then stained with uranyl acetate and lead citrate. All images were obtained with a transmission electron microscope (TECNAI10, Philips) at 80 kV.

### Immunohistochemistry

The methods of anesthetization and perfusion were the same as for transmission electron microscopy before the brains were removed. The brains were post-fixed in paraformaldehyde for longer than 18 h, and placed in a container with 30% sucrose in fixative until the tissue sank to the bottom of the container. The brains were sliced at a thickness of 20 µm. For immunohistochemistry, the sections were washed with 0.01 M PBS three times, incubated in 3% H_2_O_2_ for 15 min, washed with 0.01 M PBS three times, and then blocked with 1% bovine serum albumin and 0.4% Triton X-100 for 30 min in 37°C. The sections were incubated for 2 h with the following primary antibodies: rabbit anti-Olig2 (1∶200, Millipore), rabbit anti-Caspase 3 (1∶200, Santa Cruz) at 37°C, and then overnight at 4°C. After washing with 0.01 M PBS three times, the sections were incubated in biotin-conjugated IgG for 2 h at 37°C, washed with 0.01 M PBS three times, incubated for 1 h in ABC complex (VECTASTAIN Elite ABC Kit, Vector) at 37°C and then visualized with a chromogen solution that contained 0.05% 3′,3′-diaminobenzadine (DAB; Sigma). For immunofluorescence, H_2_O_2_ incubation step was eliminated. After incubation in primary antibody goat anti-MBP (1∶500, Santa Cruz), rabbit anti-PDGFRα (1∶200, Santcruz), goat anti-Sox10 (1∶200, Santa Cruz), mouse anti-CC-1 (1∶200, Calbiochem), the sections were incubated with Alexa Fluor 488 -conjugated donkey anti-goat or donkey anti-mouse, and 568-conjugated donkey anti-rabbit secondary antibody (1∶500), at 37°C for 2 h, and then nuclear-stained with 0.01% 4′,6′-diamidino-2-phenylindole (Sigma), and mounted onto coverslips with Glycergel mounting medium (Dako). Negative controls used PBS or polyclonal rabbit immunoglobulin G (Santa Cruz) instead of the primary antibody.

### TUNEL Staining

The tissue sections were washed for 5 min three times with 0.01 M PBS, 3% H_2_O_2_ and methanol (9∶1) for 10 min, with 0.01 M PBS for 5 min three times, and with 0.01% Triton X-100 and sodium citrate for 2 min on ice. Then sections were reacted with the TUNEL reaction mixture (In Situ Cell Death Detection Kit, Roche) for 60 min at 37°C, washed with 0.01 M PBS for 5 min three times, before being incubated with horse-radish peroxidase (POD) for 30 min at 37°C. The slides were washed with 0.01 M PBS for 5 min three times, and visualized with a chromogen solution containing DAB. The negative control had only the labeling solution instead of the TUNEL reaction mixture.

### Image Acquisition

The images were obtained with a light microscope and camera (U-ND25-2, Olympus), a fluorescence microscope (ECLIPSE 90i, Nikon), or a laser scanning confocal microscope (FV1000, Olympus) with excitation and emission wavelengths at 488 nm (Alexa Fluor 488), or 568 nm (Alexa Fluor 568), respectively, at the same exposure.

### Data Processing and Analysis

Coronal sections from +0.7 mm, +0.48 mm, +0.2 mm, –0.26mm and –0.3 mm with respect to bregma were selected according to Plate 29–48 from the mouse brain atlas [25]. The corpus callosum was analyzed in the gray areas (Figure S1).

All samples were labeled randomly, and analysis was performed using a double-blind method. The mean optical density (OD) of myelin for LFB and MBP staining, myelin in electron microscopy pictures, as well as the numbers of TUNEL+, Caspase 3+, GFAP+, PDGFRα+, Olig2+, Sox10+, CC1+ cells per mm^2^ were analyzed by Image-Pro-Plus (IPP) 6.0 software. Thirty-five samples (the sum with five coronal sections from seven mice) were selected for statistical analysis for each group.

For quantitative analysis, the groups were analyzed using one-way analysis of variance (ANOVA) followed by *Tukey’s post-hoc* test. Comparisons between two groups were made using the *Student’s t* test. The data are presented as the mean ± SEM in these groups. *P*<0.05 was used to determine significance.

## Results

### Body Weight Increased with Progesterone Treatment

**Figure 1:**
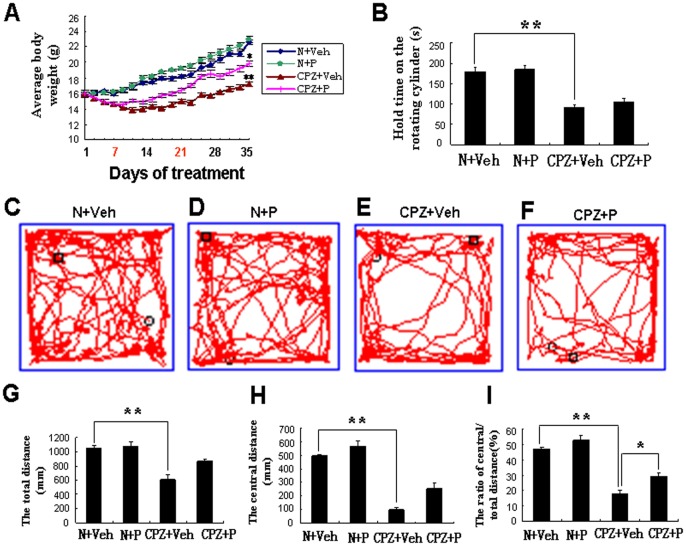
Changes in body weight, Rota-rod and open field test showed that progesterone protected cuprizone-induced mice from demyelination. (A) Body weight in N+Veh (blue), N+P (green), CPZ+Veh (red) and CPZ+P group (brown) during the 5 weeks of treatment. There was significant difference between CPZ+Veh and N+Veh, between CPZ+Veh and CPZ+P at the fifth weeks. *CPZ+P vs. CPZ+Veh, *P*<0.05; **CPZ+Veh vs. N+Veh group, *P*<0.01. Progesterone treatment was given from 7 to 21 days (red in X axis). (B) The length of time the mice stayed on the cylinder in the Rota-rod test in N+Veh, N+P, CPZ+Veh and CPZ+P group. There was no significant difference between N+Veh and N+P group (*P*>0.05), between CPZ+Veh and CPZ+P group (*P*>0.05). The trajectories traveled by the mice in the open field test showed how the mice traveled in the N+Veh (C), N+P (D), CPZ+Veh (E) and CPZ+P groups (F). The total traveled distance (G), central area traveled distance (H), and ratio of central area distance by total traveled distance (I) in these four groups. The total traveled distance was not significantly different between N+Veh and N+P group, between the CPZ+Veh and CPZ+P groups. *CPZ+P vs. CPZ+Veh, *P*<0.05; **CPZ+Veh vs. N+Veh group, *P*<0.01.

The body weights of 6-week-old, 15 to 17 g male C57BL/6 mice were recorded. In the N+Veh group, the average body weight of mice increased gradually from 15.89±0.27 g to 22.59±0.38 g at the end of 5 weeks. These mice gained 42.2% body weight during the 5 weeks (Figure 1A).

In the N+P group, the average body weight increased from 15.94±0.29 g to 22.96±0.51 g. These mice gained 44.04% body weight during the 5 weeks (Figure 1A), which was no significant difference (*P*>0.05) compared with the N+Veh group.

The average body weight increased from 15.96±0.46 g to 17.2±0.27 g at the end of 5 weeks for mice in the CPZ+Veh group, which was significantly different (*P*<0.01) than the N+Veh group. These mice only gained 7.8% body weight during the 5 weeks (Figure 1A).

In the CPZ+P group, the average weight increased from 16.01±0.46 g to 19.8±0.35 g at the end of the 5 weeks, which was significantly different (*P*<0.05) than the CPZ+Veh group (17.2±0.27 g). These mice gained 23.7% body weight during the 5 weeks (Figure 1A), which was 14.8% more than in the CPZ+Veh group.

These results showed that mice in the CPZ+Veh group gained the least body weight out of the four groups. The body weights of the progesterone treated CPZ group were significantly increased when compared with the vehicle treated CPZ group.

### The Exploratory Ability of Mice was Improved Greatly with Progesterone Treatment with No Significant Difference in Motor Coordination

Mice in the N+Veh group remained on the Rota-rod for 179.15±12.17 s at 40 rpm (Fig. 1B). In the N+P group, mice could stay on the Rota-rod for 183.21±8.76 s, which was not significantly different when compared with the N+Veh group (*P*>0.05) (Fig. 1B). However, mice in the CPZ+Veh group could only remain on the Rota-rod for 91.56±5.21 s (*P*<0.01), or approximately half the length of time when compared with the N+Veh group at the end of the 5 weeks (Fig. 1B). In the CPZ+P group, mice stayed on the Rota-rod for 104.42±9.13 s, which was also not significantly different when compared with the CPZ+Veh group (*P*>0.05) (Fig. 1B). Rota-rod analyses demonstrated that mice in the CPZ+Veh group had poorer motor coordination when compared with the N+Veh group, but there was no significant difference in motor coordination after progesterone treatment between the CPZ+P and CPZ+Veh group, and between the N+P and N+Veh groups.

The open-field test was used mainly as a measure of exploration ability. In the N+Veh group, the average value of total traveled distance was 10579.43±327.28 mm in 5 min (Fig. 1C and G), the center-area distance was 498.17±14.38 mm (Fig. 1H) by the open field test, and the ratio of center-area distance by total traveled distance was 4.71±0.13% (Fig. 1I).

In the N+P group, the average value of total distance traveled was 10784±634.57 mm in 5 min (Fig. 1D and G), and the center-area distance was 567.41±47.29 mm (Fig. 1H). The ratio of center-area distance by total traveled distance was 5.26±0.32%, and was not significantly different (*P*>0.05) when compared with the N+Veh group (Fig. 1I).

In the CPZ+Veh group, the average value of total distance traveled was 6153.47±548.74 mm in 5 min (Fig. 1E and G), and the center-area distance was 103.04±13.97 mm (Fig. 1H). The ratio of center-area distance by total traveled distance was 1.78±0.26%, and was significantly different (*P*<0.01) when compared with the N+Veh group (Fig. 1I).

In the CPZ+P group, the average value of total distance traveled was 8711.83±218.71 mm in 5 min (Fig. 1F and G), but there was no significant difference (*P*>0.05) when compared with the CPZ+Veh group (Fig. 1G). The center-area distance was 256.12±38.17 mm (Fig. 1H), the ratio of center-area distance by total distance traveled was 2.94±0.22%, which was significantly increased (*P*<0.01) when compared with the CPZ+Veh group (1.78±0.26%), but was still lower than the N+Veh group (*P*<0.05) (Fig. 1I).

Open field and Rota-rod analyses showed that the exploratory ability was poorer in the cuprizone-induced group compared with the N+Veh group, which was greatly improved in progesterone treated cuprizone group but without any significant difference in motor coordination.

### Progesterone Alleviated Demyelination of Cuprizone-induced Mice

**Figure 2:**
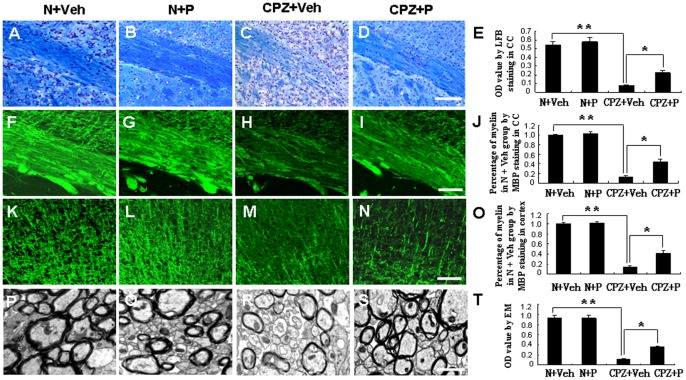
Progesterone alleviated demyelination in cuprizone-induced mouse corpus callosum and cortex by LFB, MBP staining and electron microscopy. Luxol fast blue (LFB) staining is shown in N+Veh (A), N+P (B), CPZ+Veh (C) and CPZ+P mice (D). (E) Statistical analysis of the LFB staining results according to the relative optical density. Bar = 100 µm. MBP-stained myelin is shown in the corpus callosum of N+Veh (F), N+Veh (G), CPZ+Veh (H) and CPZ+P group (I). (J) Statistical analysis by MBP staining according to the relative optical density. MBP-stained myelin is also shown in the cortex of N+Veh (K), N+Veh (L), CPZ+Veh group (M) and CPZ+P group (N). (O) Statistical analysis by MBP staining according to the relative optical density. Bar = 50 µm. Myelin was observed in the corpus callosum of N+Veh (P), N+Veh (Q), CPZ+Veh group (R) and CPZ+P treated group (S) by electron microscopy. (T) Statistical analysis by electron microscopy. Bar = 1 µm in P, Q, R and S. *CPZ+P vs. CPZ+Veh group, *P*<0.05; **CPZ+Veh vs. N+Veh group, *P*<0.01.

Blue and dense myelin by LFB staining was evident in the lateral regions of the corpus callosum in the N+Veh group (OD = 0.547±0.033) (Fig. 2A and E) and N+P group (OD = 0.579±0.048) (Fig. 2B and E). There was no significant difference in these two groups (*P*>0.05) (Fig. 2E). There was 86.5% demyelination (OD = 0.074±0.009, *P*<0.01) of the corpus callosum in the CPZ+Veh group (Fig. 2C and E) compared with the N+Veh group. In the CPZ+P group (Fig. 2D and E), there was only 58.3% demyelination (OD = 0.228±0.021, *P*<0.05) compared with the N+Veh group.

Dense and deeply-stained myelin by MBP immunofluorescence staining was evident in the lateral regions of the corpus callosum in the N+Veh (Fig. 2 F and J) and N+P group (Fig. 2G and J), and also in the cortex in the N+Veh (Fig. 2 K and O) and N+P group (Fig. 2L and O). There was no significant difference of myelin between in the N+Veh and N+P group in the lateral regions of the corpus callosum (Fig. 2J) and in the cortex (Fig. 2O) in these two groups (*P*>0.05). But there was less myelin in the lateral regions of the corpus callosum (13.47±0.24%, *P*<0.01) (Fig. 2H and J) and in the cortex (14.29±0.27%, *P*<0.01) (Fig. 2M and O) in the CPZ+Veh group than in the N+Veh group. In the CPZ+P group, thicker myelin was observed in the corpus callosum (43.46±0.73%, *P*<0.05) (Fig. 2I and J), also in the cortex (41.25±0.58%, *P*<0.05) (Fig. 2N and O) compared with the CPZ+Veh group.

Dense and thick myelin was showed in the corpus callosum of the N+ Veh group (Fig. 2P and T) and N+ P group (Fig. 2Q and T) by electron microscopy. There was no significant difference (*P*>0.05) of myelin between in the N+Veh and N+P group (Fig. 2T). However, in the CPZ+Veh group (Fig. 2R and T), there was only 13.21±0.98% myelin (*P*<0.01) compared with in the N+ Veh group. In the CPZ+P group (Fig. 2S and T), there was 38.78±1.47% myelin (*P*<0.05) compared with in the N+Veh group.

These results indicate severe demyelination in the vehicle-treated cuprizone group and a mitigation of demyelination with progesterone treatment.

### Progesterone Reduced the Number of TUNEL-positive Apoptotic Cells in the Corpus Callosum and Caspase-3-positive Cells in the Cortex of Cuprizone-induced Mice

**Figure 3:**
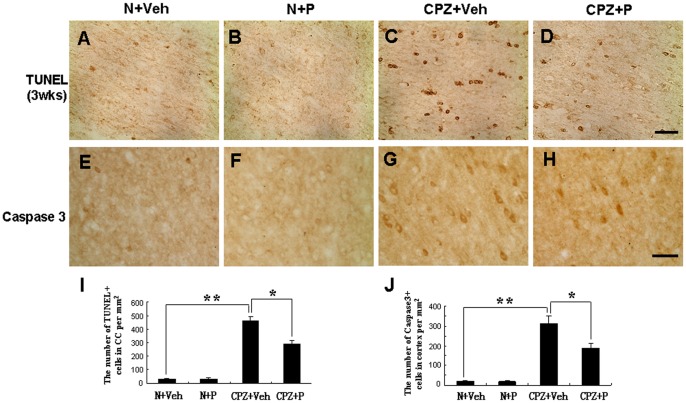
Progesterone rescued the loss of TUNEL-positive cells in the corpus callosum and Caspase3-positive cells in the corpus callosum of cuprizone-induced mice. TUNEL-positive cells were observed in the corpus callosum at 3 weeks in the N+Veh (A), N+P (B), CPZ+Veh (C), and CPZ+P group (D). (E) Statistical analyses showed that TUNEL-positive cells decreased in the CPZ+P group compared with the CPZ+Veh group. Bar = 50 µm. Caspase3-positive cells were observed in N+Veh group (F), N+P group (G), CPZ+Veh group (H), and CPZ+P group (I) in the cortex of mice. (J) Statistical analyses showed that Caspase3-positive cells decreased in the CPZ+P group compared with the CPZ+Veh group. Bar = 50 µm. *CPZ+P vs. CPZ+Veh group, *P*<0.05; **CPZ+Veh vs. N+Veh group, *P*<0.01.

We compared the number of TUNEL positive cells in the corpus callosum from one to five weeks. There was a significant difference between the CPZ+Veh and CPZ+P group at the end of 3 weeks. A few TUNEL-positive cells were observed in the corpus callosum in the N+Veh group (30.45±8.14 per mm^2^) (Fig. 3A and I) and N+P group (27.93±10.21 per mm^2^) (Fig. 3B and I) at the end of 3 weeks. The number of TUNEL-positive cells increased significantly in the CPZ+Veh group (458.29±34.78 per mm^2^, *P*<0.01) (Fig. 3C and I) and decreased after progesterone treatment in the CPZ+P group (289.45±28.79 per mm^2^, *P*<0.05) (Fig. 3D and I).

Caspase3+ cells were mainly located in the gray matter, with only a few Caspase3+ cells found in the corpus callosum. There were very few Caspase3+ cells in the cortex of mice in the N+Veh group (17.2±3.1) (Fig. 3E and J) and the N+P group (18.4±2.6) (Fig. 3F and J). After 5 weeks of cuprizone treatment, mice exhibited increased Caspase3+ cells (313.2±35.6, *P*<0.01) per mm^2^ in the CPZ+Veh group (Fig. 3G and J) compared with the N+Veh group. In the CPZ+P group (Fig. 3H and J), the number of Caspase3+ cells (185.7±28.4, *P*<0.05) per mm^2^ was significantly decreased compared with the CPZ+Veh group.

These results suggest that progesterone reduced the number of TUNEL-positive apoptotic cells in the corpus callosum and the number of Caspase3+ cells in the cortex of cuprizone-induced mice.

### Progesterone Reduced the Number of Astrocytes in Cuprizone-induced Mice

**Figure 4:**
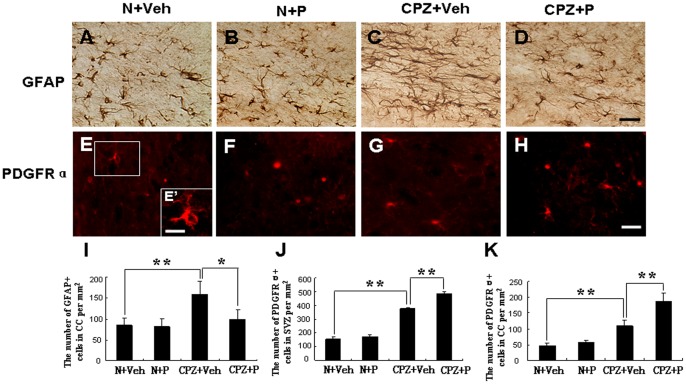
Progesterone reduced the increase of GFAP-positive cells in the corpus callosum and increased PDGFRα-positive OPCs. GFAP-positive cells were observed in the N+Veh group (A), N+P group (B), CPZ+Veh group (C), and CPZ+P group (D) in the corpus callosum of mice. (I) Statistical analyses showed that GFAP-positive cells decreased in the CPZ+P group compared with the CPZ+Veh group. Bar = 50 µm. *CPZ+P vs. CPZ+Veh group, *P*<0.05; **CPZ+Veh vs. N+Veh group, *P*<0.01. PDGFRα-positive OPCs were observed in the SVZ of mice in the N+Veh group (E and E’), N+P group (F), CPZ+Veh group (G), and CPZ+P group (H). (J) Statistical analyses showed that PDGFRα-positive cells increased in the CPZ+P group compared with the CPZ+Veh group. The number of PDGFRα-positive OPCs per mm^2^ is seen in the corpus callosum (K). Bar = 50 µm in A–H; Bar = 10 µm in E’. **CPZ+Veh vs. N+Veh group; CPZ+P vs. CPZ+Veh group, *P*<0.01.

The number of GFAP-positive astrocytes was 84.27±17.48 per mm^2^ in the corpus callosum of the N+Veh group (Fig. 4A and I), was 81.17±19.24 per mm^2^ in the N+P group (Fig. 4B and I), which was significantly increased in the CPZ+Veh group (158.42±31.78, *P*<0.01) (Figure. 4C and I) compared with N+Veh group. In the CPZ+P group, the number of GFAP-positive astrocytes significantly decreased to 99.22±22.30 (*P*<0.05) (Figure. 4D and I) compared with the CPZ+Veh group. These results suggest that progesterone reduced the number of astrocytes in cuprizone-induced mice.

### Progesterone Increased the Number of OPCs and Rescued the Loss of Oligodendroglia Cells in Cuprizone-induced Mice

PDGFRα was used as one of the OPC markers. Changes in OPC number, which subsequently migrate to the corpus collosum, were observed in the subventricular zone (SVZ). The number of PDGFRα+ OPCs was 156.18±14.19 per mm^2^ in the SVZ of the N+Veh group (Fig. 4E and J), which was slightly higher in the N+P group (174.35±12.87 per mm^2^; Fig. 4F and J), and significantly higher in the CPZ+Veh group (376.47±10.19 per mm^2^ (*P*<0.01) (Fig. 4G and J). The number of cells in the SVZ increased to 484.18±14.62 per mm^2^ (*P*<0.01) in the CPZ+P group (Fig. 4H and J). The number of PDGFRα+ OPCs was 47.21±7.98 per mm^2^ in the corpus callosum in the N+Veh group; however, there was no obvious change (56.74±18.43 per mm^2^, *P*>0.05) in the N+P group. The number of cells increased to 109.4±18.7 per mm^2^ (*P*<0.01) in the CPZ+Veh group and was significantly higher in the CPZ+P group 187.4±27.1 per mm^2^ (*P*<0.01) (Fig. 4K). These results showed that progesterone increased the number of PDGFRα+ OPCs in cuprizone-induced mice.

**Figure 5:**
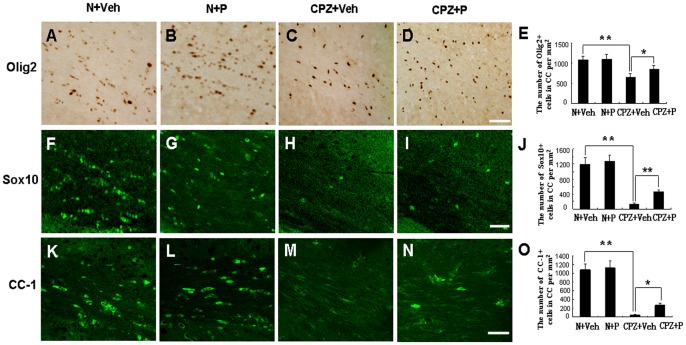
Progesterone rescued the loss of oligodendroglial cells in the cuprizone-induced mice. Olig2-positive cells were observed in the corpus callosum of N+Veh (A), N+P (B), CPZ+Veh (C), and CPZ+P group (D). (E) Statistical analyses showed that Olig2-positive cells increased in the CPZ+P group compared with the CPZ+Veh group. Bar = 50 µm. *CPZ+P vs. CPZ+Veh group, *P*<0.05; **CPZ+Veh vs. N+Veh group, *P*<0.01. Sox10-positive cells were observed in the corpus callosum of mice in N+Veh (F), N+P (G), CPZ+Veh (H), and CPZ+P group (I). (J) Statistical analyses showed that the number of Sox10-positive cells increased in the CPZ+P group compared with the CPZ+Veh group. Bar = 50 µm. **CPZ+Veh vs. N+Veh, CPZ+ P vs. CPZ+Veh, *P*<0.01. CC-1+ mature oligodendrocytes were observed in the corpus callosum of mice in N+Veh group (K), N+P group (L), CPZ+Veh group (M), and CPZ+P group (N). (O) Statistical analysis showed that CC-1-positive cells increased in the CPZ+P group compared with the CPZ+Veh group. Bar = 50 µm. *CPZ+P vs. CPZ+Veh group, *P*<0.05; **CPZ+Veh vs. N+Veh group, *P*<0.01.

Olig2 is one of the most commonly used markers of oligodendroglial cells. The number of Olig2-positive cells was 1047.13±97.65 per mm^2^ in the corpus callosum in the N+Veh group (Fig. 5A and E). In the N+P group, the number of cells was 1124.36±104.27 per mm^2^ (Fig. 5B and E). There was no significant difference between these two groups (*P*>0.05) (Fig. 5E). In the CPZ+Veh group, the number of cells was 652.47±92.46 per mm^2^ (Fig. 5C and E), which was lower than in the N+Veh (*P*<0.01). In the CPZ+P group, the number of cells was 853.64±95.48 per mm^2^ (Fig. 5D and E), which was higher than in the CPZ+Veh group (*P*<0.05). These results showed that progesterone rescued the loss of Olig2-positive cells in the corpus callosum of cuprizone-induced mice.

Sox10 expression in oligodendroglial cells mediates terminal differentiation of myelin-forming oligodendrocytes [26]. Sox10-positive cells were observed in the corpus callosum of the N+Veh group (1188.36±184.31 per mm^2^) (Fig. 5F and J), which was not significantly different from the N+P group (1266.32±184.78 per mm^2^, *P*>0.05) (Fig. 5G and J). The number of cells reached 126.33±38.85 per mm^2^ in the CPZ+Veh group (Fig. 5H and J), and this number significantly increased (456.78±58.47 per mm^2^, *P*<0.01) in the CPZ+P group (Fig. 5I and J) when compared with the CPZ+Veh group, but these were still fewer than the N+Veh group. These results show that progesterone rescued the loss of Sox10-positive cells in the corpus callosum of cuprizone-induced mice.

CC-1, a marker of mature oligodendrocytes, was also monitored in this study. In the N+Veh group, the number of CC-1-positive mature oligodendrocytes was 1047.27±134.46 per mm^2^ (Fig. 5K and O) in the corpus callosum. In the N+P group, levels reached 1121.34±157.63 per mm^2^ (Fig. 5L and O), and there was no significant difference when compared with the N+Veh group (*P*>0.05). These mice exhibited very few CC-1+ mature oligodendrocytes (45.72±12.83 per mm^2^, *P*<0.01) (Fig. 5M and O) compared with the N+Veh or N+P group. In the CPZ+P group, the number of oligodendrocytes significantly increased (267.34±46.48 per mm^2^, *P*<0.05) (Fig. 5N and O) compared with the CPZ+Veh group. Thus, progesterone rescued the loss of CC-1+ mature oligodendrocytes in the corpus callosum of cuprizone-induced mice.

## Discussion

### Changes in Body Weight and Behavioral Analysis were Consistent with the Protective Function of Progesterone

Normal female rats treated with progesterone showed an obvious increase of the body weight and composition. But male rats treated with progesterone showed no changes other than a small gain of water [27]. In our study, all the mice are male. There is no significant increase in N+P group compared with N+Veh group, while mice gained body weight in CPZ+P group compared with CPZ+Veh group. The reason which progesterone increased body weight of cuprizone induced mice may be mainly due to protective function of progesterone on cuprizone intoxication.

The ratio of the center-area traveled distance by the total traveled distance was evaluated as a measure of exploration ability in the open-field test [3], [28]. The significant decrease reflected poorer exploration ability after demyelination. Previous study showed that the cuprizone treated mice had poorer motor coordination than normal mice by Rota-rod analysis [3], [28], our result also showed poorer motor coordination of the cuprizone treated mice, but there was no significant difference after progesterone treatment. Open field analysis indicated that there was marked improvement in the exploration ability of the CPZ+P group compared with the CPZ+Veh group, but there was no significant difference in the N+P group compared with the N+Veh group. These results indicate that progesterone which increased body weight and protected against behavioral deficits are consistent with the protective effects of progesterone against demyelination.

### Progesterone Inhibiting Astrogliosis was Consistent with the Protective Effect on Demyelination

One of the first responses following cuprizone treatment is activation of reactive astrocytes during the 1st and 2nd weeks after treatment [3], [22]. Our results revealing an increase in astrogliosis in cuprizone fed mice are consistent with these results. Acs et al showed that the combined application of estradiol and progesterone can protect the brain from demyelination with increasing microgliosis and astrogliosis in cuprizone-induced mice [24]. Single administration of progesterone with low doses (14 days of 0.5mg/kg) resulted in moderate prevention of demyelination in the corpus callosum, but did not show a significant up-regulation of GFAP mRNA expression [24]. Large doses of progesterone inhibit astrocyte activation after spinal cord injury [29], our results showed that large doses of progesterone prevented astrogliosis, which is consistent with this study, and provides obvious protection against demyelination.

### Progesterone Alleviated Demyelination with an Increase in the Number of OPCs

There was severe demyelination in the corpus callosum after the fifth week of cuprizone treatment [22], [24]. In this study, progesterone treatment alleviated demyelination as assessed by electron microscopy following LFB and MBP staining. These results are consistent with previous reports that progesterone had a protective effect on demyelination [2], [4], [7], [24].

OPCs proliferated and migrated from the SVZ and fornix, and then accumulated within the cuprizone induced demyelinated regions [22]. Progesterone has been shown to protect the brain from demyelination, and affect early proliferation OPCs and late remyelination after spinal cord injury [29]. In this study, our results showed that the number of PDGFRα-positive OPCs increased in the corpus callosum and the SVZ of mice in the CPZ+P group when compared with mice in the CPZ+Veh group. These results are consistent with the hypothesis that progesterone increases the number of OPCs.

### Progesterone Rescued Oligodendroglial Cell Death

Oligodendroglial cell death occurs gradually after a few days at the beginning of cuprizone treatment and before demyelination is visible in the fifth week [22], [30], [31]. In this study, progesterone was given to mice for 2 weeks from the 2nd week to the end of the 3rd week of cuprizone treatment. The timing of progesterone treatment was consistent with the peak period of oligodendroglial cell death [22, 30 31]. Our results showed that progesterone reduced the number of TUNEL-positive and Caspase-3-positive cells, rescued the loss of Olig2-positive oligodendroglial cells, and induced an increase in the proliferation of OPCs in cuprizone-induced mouse. These results indicated that progesterone may promote OPC differentiation into oligodendroglial cells. However, proliferating OPCs have difficulty differentiating into mature oligodendrocytes and re-forming myelin in the cuprizone induced mouse brain [30], [31]. CC-1 is a marker of mature oligodendrocytes and Sox10 mediates terminal differentiation of myelin-forming oligodendrocytes [26]. Therefore in this study, the loss of Sox10 and CC-1 expression in oligodendrocytes is only slightly rescued by progesterone. These results indicated that progesterone reduced the number of apoptotic cells and rescued oligodendroglial cell death.

### The Doses of Cuprizone and Progesterone Treatment

Obvious demyelination was observed after 0.2% or 0.3% (w/w) cuprizone, which was mixed in the diets to feed the mice for 6 weeks. Mice are poisoned and can die with diets containing 0.6% (w/w) cuprizone, according to previous studies [3], [22], [24]. Low doses (14 days of 0.5mg/kg) of progesterone resulted in moderate prevention of demyelination in the corpus callosum [22]. Large doses (14 days of 5 mg/kg) of progesterone significantly limited the damage to the CNS during the first few hours to days after injury, reduced loss of neural cells, and improved functional recovery [29]. In this study, we used large doses (14 days of 5 mg/kg) of progesterone and observed the protective effect on demyelination in the cuprizone-induced model.

In conclusion, our results showed the protective effect of progesterone on neural behavioral deficits by detecting changes in body weight, and activity during the open field and Rota-rod tests. In addition, progesterone had a protective effect against demyelination, with reduced degeneration of oligodendroglial cells in the cuprizone-injured mouse brain. These results indicate that progesterone can be used to protect against demyelination of the brain. However, the protective mechanism of progesterone still requires further research.

## Supporting Information

Figure S1 **Ideograph of coronal section from +0.7 mm with respect to bregma in the adult mouse brain.** Gray region represents the corpus callosum. AC: the anterior commissure; LV: lateral ventricle.
(TIF)
