# Supplementary figures and images for: Progesterone Alleviates Neural Behavioral Deficits and Demyelination with Reduced Degeneration of Oligodendroglial Cells in Cuprizone-Induced Mice
Source: PLoS One. 2013 Jan 24;8(1):e54590. doi: 10.1371/journal.pone.0054590 (PMC3554738; doi:10.1371/journal.pone.0054590)

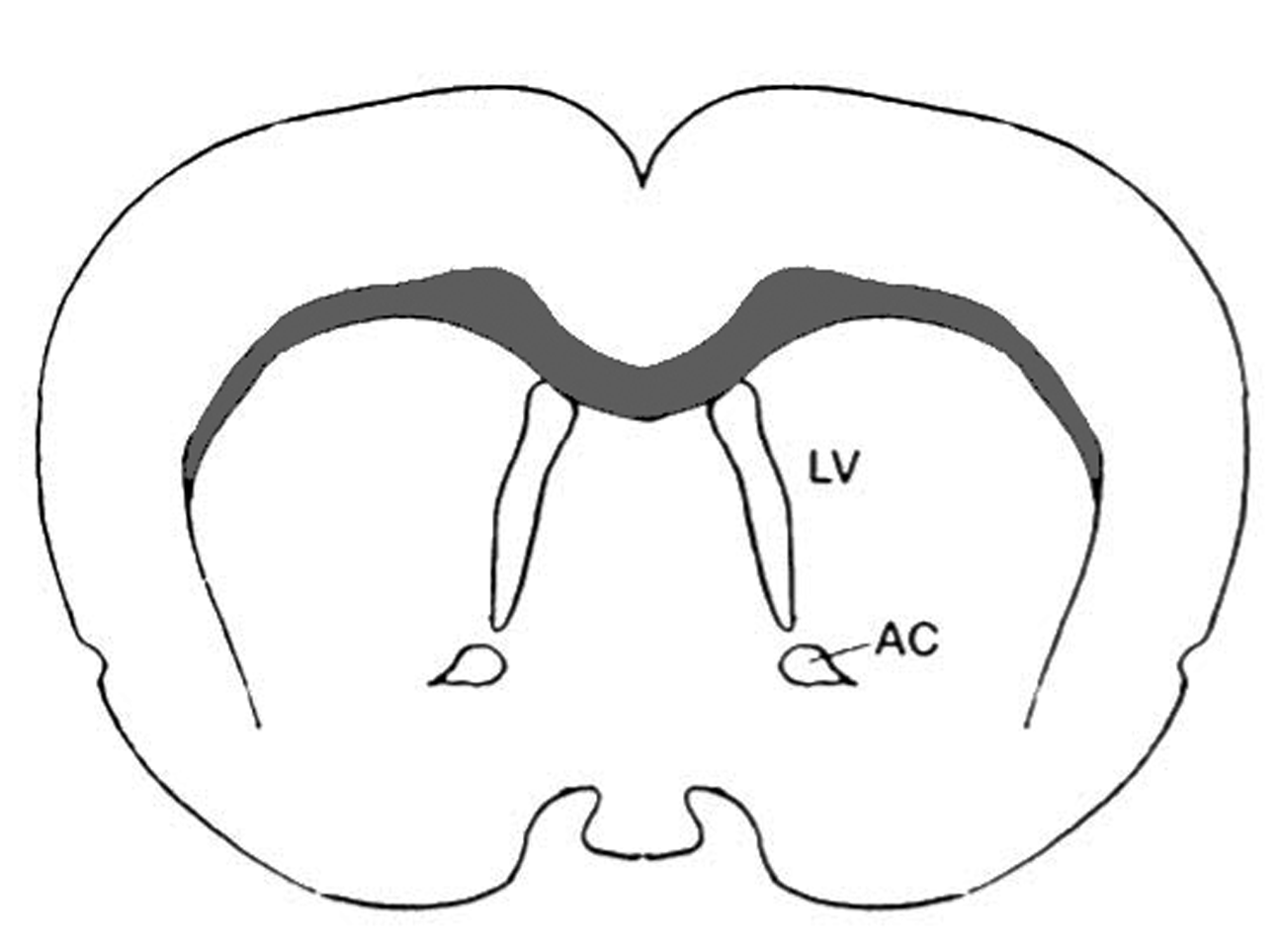

Supplement: Figure S1 — Ideograph of coronal section from +0.7 mm with respect to bregma in the adult mouse brain. Gray region represents the corpus callosum. AC: the anterior commissure; LV: lateral ventricle. (TIF) [file pone.0054590.s001.tif]
